# Meal-feeding promotes skeletal growth by ghrelin-dependent enhancement of growth hormone rhythmicity

**DOI:** 10.1172/JCI189202

**Published:** 2025-04-01

**Authors:** Amanda K.E. Hornsby, Richard C. Brown, Thomas W. Tilston, Harry A. Smith, Alfonso Moreno-Cabañas, Bradley Arms-Williams, Anna L. Hopkins, Katie D. Taylor, Simran K.R. Rogaly, Lois H.M. Wells, Jamie J. Walker, Jeffrey S. Davies, Yuxiang Sun, Jeffrey M. Zigman, James A. Betts, Timothy Wells

**Affiliations:** 1 School of Biosciences, Cardiff University, Cardiff, United Kingdom.; 2 Institute of Life Sciences, School of Medicine, Swansea University, Swansea, United Kingdom.; 3 Centre for Nutrition, Exercise and Metabolism, Department for Health, University of Bath, Bath, United Kingdom.; 4 Department of Mathematics and Statistics, University of Exeter, Exeter, United Kingdom.; 5 Department of Nutrition, Texas A&M University, College Station, Texas, USA.; 6 UT Southwestern Medical Center, Dallas, USA.

**Keywords:** Endocrinology, Metabolism, Bone development

## Abstract

The physiological effect of ultradian temporal feeding patterns remains a major unanswered question in nutritional science. We have used automated and nasogastric feeding to address this question in male rodents and human volunteers. While grazing and meal-feeding reduced food intake in parallel (compared with ad libitum–fed rodents), body length and tibial epiphysial plate width were maintained in meal-fed rodents via the action of ghrelin and its receptor, GHS-R. Grazing and meal-feeding initially suppressed elevated preprandial ghrelin levels in rats, followed by either a sustained elevation in ghrelin in grazing rats or preprandial ghrelin surges in meal-fed rats. Episodic growth hormone (GH) secretion was largely unaffected in grazing rats, but meal-feeding tripled GH secretion, with burst height augmented and 2 additional bursts of GH per day. Continuous nasogastric infusion of enteral feed in humans failed to suppress circulating ghrelin, producing continuously elevated circulating GH levels with minimal rhythmicity. In contrast, bolus enteral infusion elicited postprandial ghrelin troughs accompanied by reduced circulating GH, with enhanced ultradian rhythmicity. Taken together, our data imply that the contemporary shift from regular meals to snacking behavior may be detrimental to optimal skeletal growth outcomes by sustaining circulating ghrelin at levels associated with undernourishment and diminishing GH pulsatility.

## Abstract 2

Eating regular meals activates the ghrelin-growth hormone system in male rodents and humans, the additional bursts of growth hormone secretion accelerating skeletal growth in rodents.

## Graphical abstract

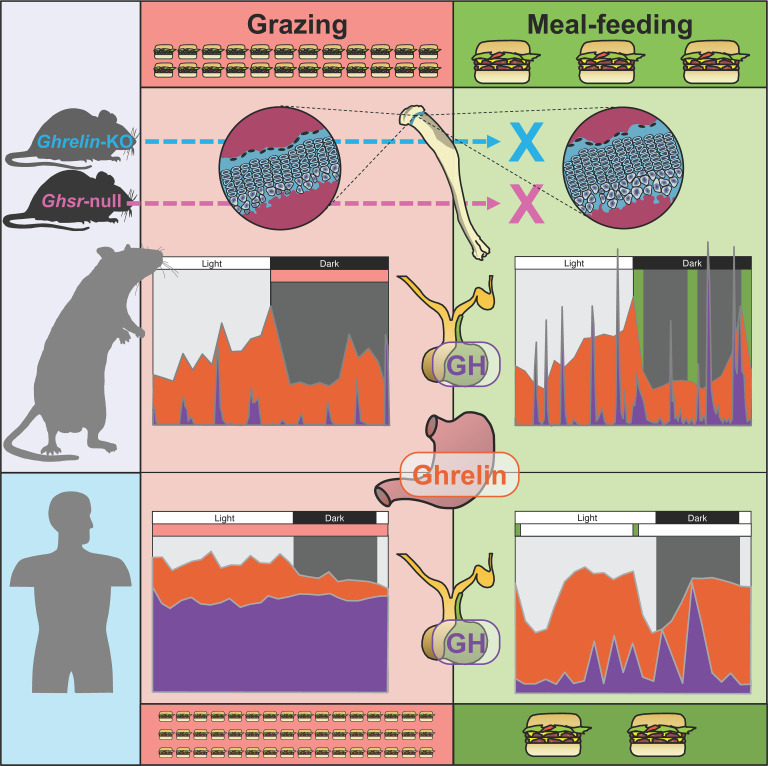

## Introduction

It is well established that circadian feeding patterns influence a wide range of physiological outcomes, but while epidemiological evidence is emerging for a potential effect of more frequent, ultradian feeding rhythms (1, 2), this aspect of chrononutrition has not been systematically explored (3, 4).

Epidemiological studies suggest associations between ultradian feeding patterns and multiple physiological variables, such as food choice (5), energy intake (6), and metabolic outcome (7, 8). Not everyone agrees (9), and this controversy arises in part from the well-recognized inaccuracy of self-reported food intake (10, 11) and the potential distortion of participant attrition (12). While the development of mobile Apps may improve reporting accuracy (13, 14), compelling evidence will only emerge from laboratory-based studies in which patterned food intake is fully controlled.

Despite their predominantly nocturnal feeding behavior, a number of approaches have been developed to study contemporary human feeding patterns in rodents (15), including the ClockLab (16), BioDAQ (17), and SnackClock (18) systems, which deliver pelleted food in user-defined patterns. However, these systems are unable to deliver the smoothed access required to study snacking/grazing behavior and are compromised by the propensity of laboratory rodents, especially female mice, to hoard food in the home cage (19, 20), thereby thwarting researcher-imposed control.

We have taken 2 approaches to overcome these limitations. Firstly, we utilized a modified comprehensive lab animal monitoring system (CLAMS) to deliver a crushed diet in consistent, reproducible temporal patterns for rats and mice (21), with the combined use of automated serial blood sampling in rats enabling us to characterize the effect of these patterns on spontaneous hormone rhythmicity. Second, we complemented this approach in human volunteers with patterned nasogastric delivery of enteral feed coupled with serial blood sampling (22).

We report here the effect of 2 specific feeding patterns — nocturnal grazing and nocturnal meal-feeding — on skeletal growth in male rats and mice, including those with a null allele for ghrelin (23) or transcriptional blockade of the growth hormone secretagog receptor (GHSR) (24), the cognate receptor for ghrelin. We also report the effect of these feeding patterns on the temporal secretion of ghrelin and growth hormone (GH) in rats alongside the effect of continuous or bolus infusions of enteral feed on ghrelin and GH levels in men.

## Results

### Study 1: Meal-feeding protects skeletal growth in male rats.

**Figure 1:**
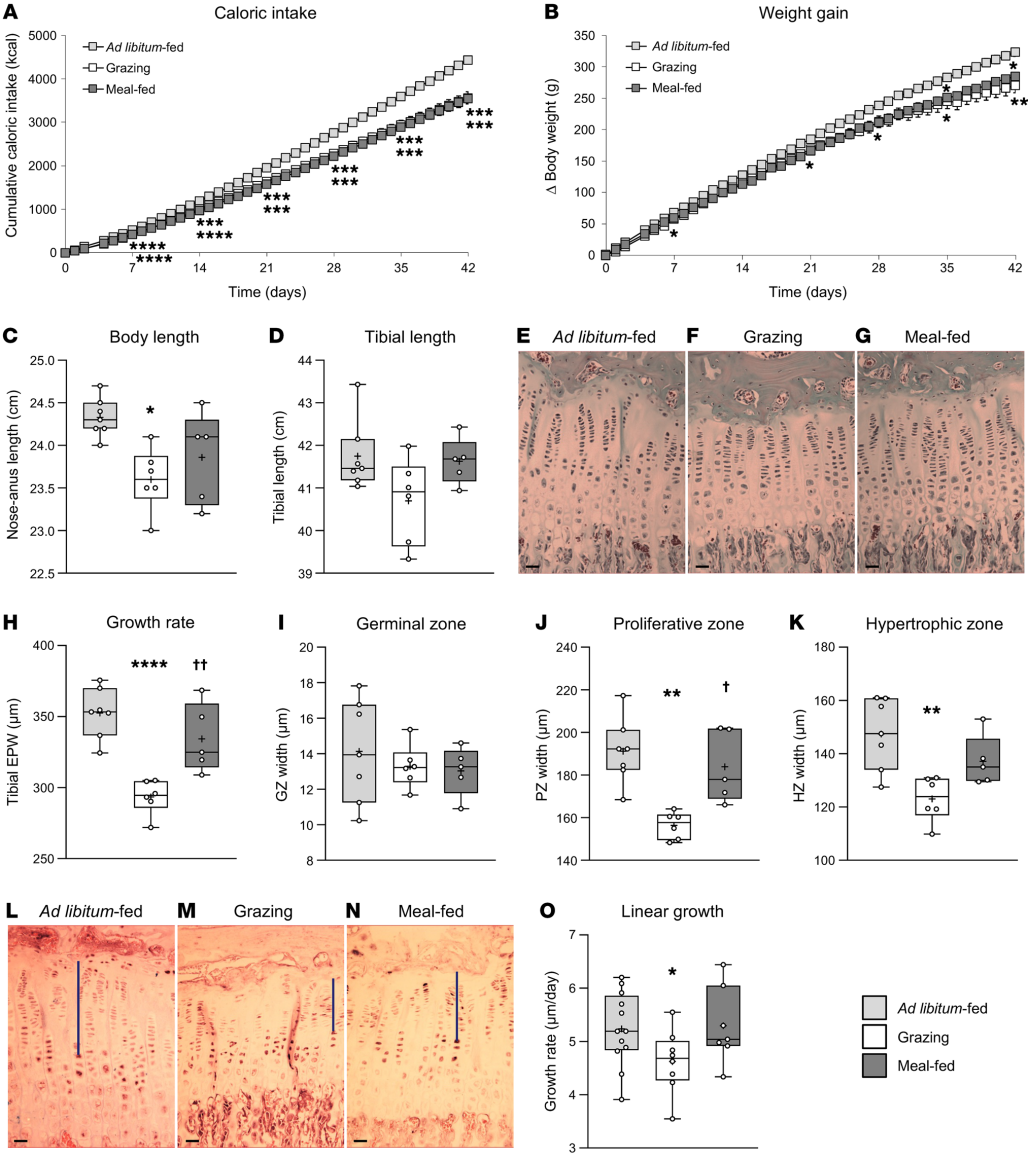
Meal-feeding protects skeletal growth (studies 1 and 2). Cumulative caloric intake (**A**), BW gain (**B**), body length (**C**), tibia length (**D**), tibia EPW (**H**), and germinal zone (GZ) (**I**), proliferative zone (PZ) (**J**), and hypertrophic zone (HZ) (**K**) widths (in Masson’s trichrome–stained sections **E**–**G**; scale bars: 20 μm) in male rats receiving standard chow in either ad libitum (light gray symbols/bars), grazing (white symbols/bars), or meal-feeding (dark gray symbols/bars) patterns for 6 weeks. In addition, the linear growth rate (**O**) was measured in tibia sections (**L**–**N**) stained for BrdU (dark nuclei; scale bars: 20 μm; blue bars indicate the distance from the GZ to the first BrdU-labeled nucleus in the column) in a separate cohort of rats subjected to these feeding patterns for 3 weeks. Data shown are the mean ± SEM (**A** and **B**); box-and-whisker plots (**C**, **D**, **H**–**K**, and **O**) show the median line, mean (+), upper and lower quartile range (bars), data range (whiskers), and individual data points. *n* = 7 (ad libitum–fed, **A**–**K**), *n* = 6 (grazing, **A**–**K**), *n* = 5 (meal-fed, **A**–**K**), *n* = 12 (ad libitum–fed, **O**), and *n* = 8 (grazing and meal-fed, **O**) with statistical comparisons performed by 1-way ANOVA and Bonferroni’s selected-pairs post hoc test. *P < 0.05, **P < 0.01, ***P < 0.001, and ****P < 0.0001 versus ad libitum–fed; ^†^*P* < 0.05; ^††^*P* < 0.01 versus grazing.

To characterize the effect of temporal feeding patterns, male rats received standard chow in a grazing (permitted access to set small amounts every 30 minutes during the dark phase [1800–0600 hours] or meal-feeding [three 1-hour periods of ad libitum access at the beginning [1800 hours], middle [2330 hours], and end [0500 hours] of the dark phase) pattern for 6 weeks. Both patterns reduced cumulative caloric intake by 20% compared with ad libitum–fed rats (Figure 1A; *P* = 0.0001 and 0.0002, respectively). Since caloric intake did not differ between grazing and meal-fed rats at any time point (day 42 cumulative caloric intake: 3,560 ± 346 kcal [grazing]; 3,460 ± 75 kcal [meal-fed]; *P* > 0.999), the differences in physiological endpoints resulted from these patterns of feeding.

BW gain was reduced by 16% and 12% (vs. ad libitum–fed rats) in grazing (*P* = 0.0014) and meal-fed (*P* = 0.0236) rats, respectively (Figure 1B) but was not different between grazing and meal-fed cohorts. Body length was reduced by 3% in grazing rats (*P* = 0.0108 vs. ad libitum–fed rats), but not in meal-fed animals (Figure 1C). Tibial lengths showed a similar pattern, with mean length in grazing rats being 97% of that in ad libitum–fed animals, but this was not significantly different (*P* = 0.1129; Figure 1D). However, tibial epiphyseal plate width (EPW; Figures 1E–G) (an accurate index of skeletal growth rate) was reduced by 17% for grazing rats (*P* = 0.0001 vs. ad libitum–fed; Figure 1H), whereas the EPW of meal-fed rats was unaffected (*P* = 0.3299 vs. ad libitum–fed; *P* = 0.0064 vs. grazing). This reduction in EPW in grazing rats was largely due to 18% and 17% reductions in proliferative (*P* = 0.0010; Figure 1J) and hypertrophic zone widths (*P* = 0.0034; Figure 1K), with the germinal zone width being unaffected (Figure 1I).

### Study 2: Grazing reduces the rate of chondrocyte migration.

To determine whether these changes in skeletal growth are reflected in chondrocyte migration, 3 cohorts of rats were treated as in study 1, with BrdU given to “birth date” dividing cells 5 days prior to 3 weeks of grazing or meal-feeding. Caloric intake and weight gain paralleled those seen after 3 weeks in study 1 (Supplemental Table 1; supplemental material available online with this article; https://doi.org/10.1172/JCI189202DS1). Although tibia length was not significantly affected after 3 weeks of patterned feeding, the tibia EPW was reduced in grazing rats by 18% (*P* = 0.003 vs. ad libitum–fed), and this was reflected in 20% and 18% reductions in the width of the proliferative and hypertrophic zones (*P* = 0.010; *P* = 0.041; Supplemental Table 1). The longitudinal growth rate (distance from the proximal surface of the germinal zone to the first BrdU^+^ nucleus/[days since BrdU injection] since BrdU injection; Figure 1, L–N) was reduced by 16% in grazing rats (*P* = 0.0099 vs. ad libitum–fed; Figure 1O). Neither the total EPW, the zone widths, nor the longitudinal growth rate were affected in meal-fed rats (Supplemental Table 1 and Figure 1O).

### Study 3: The growth effects of meal-feeding and grazing are reversed in ghrelin-KO mice.

**Figure 2:**
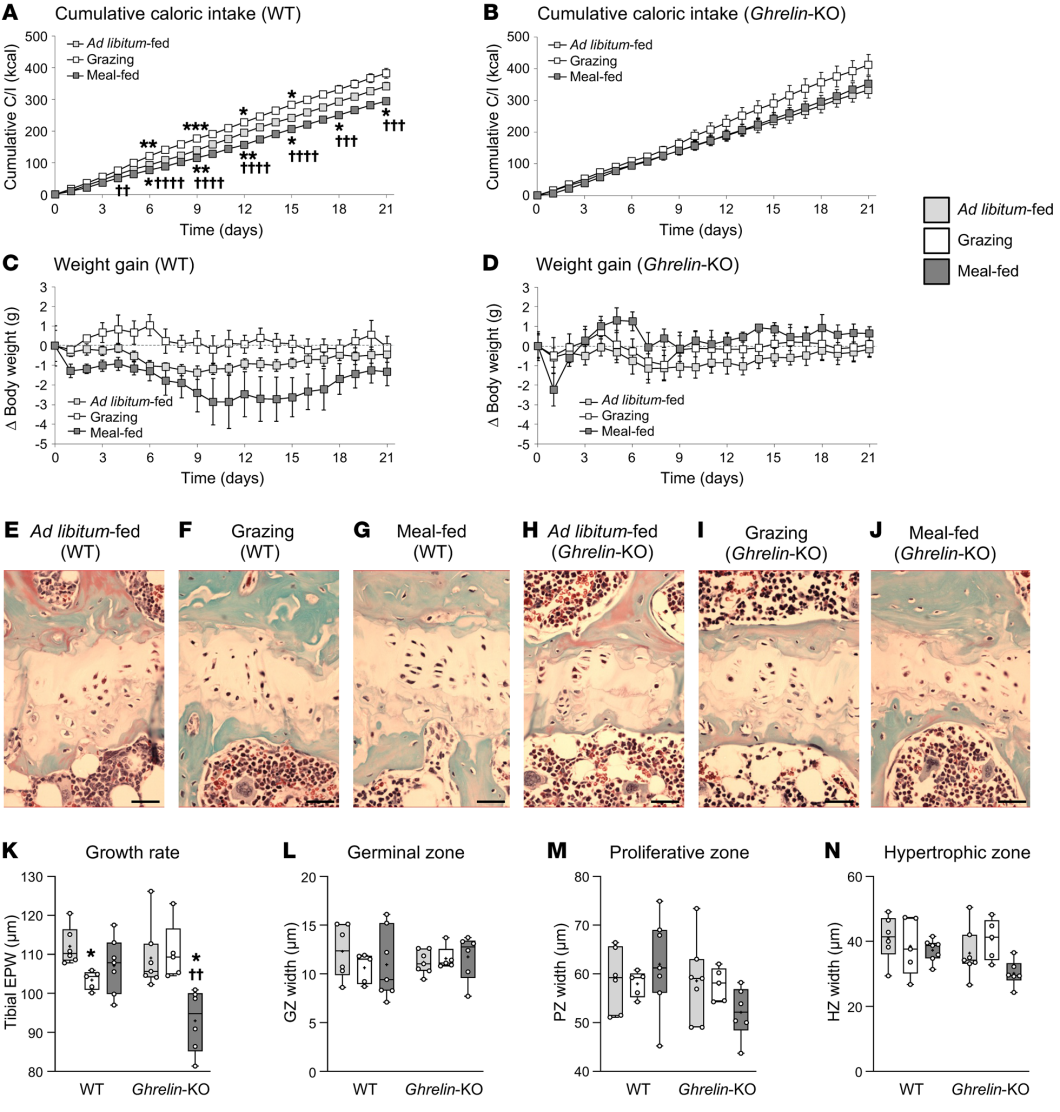
Meal-feeding promotes skeletal growth via a ghrelin-dependent mechanism. Cumulative caloric intake (C/I) (**A** and **B**), BW gain (**C** and **D**), tibia EPW (**K**), and germinal zone (**L**), proliferative zone (**M**), and hypertrophic zone (**N**) widths (measured in Masson’s trichrome–stained sections in **E**–**J**; scale bars: 20 μm), in 6-month-old male *ghrelin*-KO mice (*Ghr-*KO) (**B**, **D**, and **H**–**J**) and their WT male littermates (**A**, **C**, and **E**–**G**) fed a standard, nonpurified rodent diet (13.9% AFE fat) in either ad libitum–feeding (light gray symbols/bars), grazing (white symbols/bars), or meal-feeding (dark gray symbols/bars) patterns for 3 weeks. Data shown are the mean ± SEM (**A**–**D**), with box-and-whisker plots (**K**–**N**) showing the median line, mean (+), upper and lower quartile range (bars), data range (whiskers), and individual data points (*n* = 5, WT grazing, *ghrelin*-KO grazing; *n* = 6, WT ad libitum–fed, *ghrelin*-KO meal-fed; *n* = 7, WT meal-fed, *ghrelin*-KO ad libitum–fed). Statistical comparisons were performed by 1-way ANOVA with Bonferroni’s selected-pairs post hoc test. *P < 0.05, **P < 0.01, and ***P < 0.001 versus ad libitum–fed males (same genotype); ^††^*P* < 0.01, ^†††^*P* < 0.001, and ^††††^*P* < 0.0001 versus grazing males (same genotype).

Given that ghrelin secretion is regulated by feeding events (25–27) and its biological activity is pattern dependent (28, 29), we investigated the role of ghrelin in these feeding pattern–induced changes in *ghrelin*-KO mice. Since our CLAMS system is designed for rats, we were constrained to use larger (6-month-old) mice in this study and allowed more generous grazing (see Methods). In this context, grazing mice consumed 30% more calories than did meal-fed mice (*P* = 0.0004; Figure 2A), the latter consuming 14% fewer calories than ad libitum–fed mice (*P* = 0.032; Figure 2A). These effects were abolished in *ghrelin*-KO mice, due largely to increased consumption by the meal-fed animals (Figure 2B). As expected for adult mice, these feeding patterns had little effect on weight gain (Figure 2, C and D), body length, or any of the organ weights measured (Supplemental Table 2). Nevertheless, despite the less organized appearance of epiphyseal plates in older animals (Figure 2, E–J) and the increased caloric intake in grazing WT mice, the tibial EPW was still reduced by 8% (*P* = 0.030; Figure 2K), with no significant effect on EPW in meal-fed mice. Remarkably, this response was not only abolished in *ghrelin*-KO mice but was reversed, with grazing *ghrelin*-KO mice showing no reduction in EPW (*P* = 0.909), and the EPW in meal-fed *ghrelin*-KO mice being 12% (*P* = 0.012) and 16% (*P* = 0.002) lower than that in ad libitum–fed and grazing mice, respectively Figure 2K). Individual zone widths were not significantly affected (Figure 2L–N). Although. Although mean plasma insulin-like growth factor 1 (IGF-1) levels in grazing WT males were only 70% of the levels in ad libitum–fed mice, mean IGF-1 values were not significantly different (Supplemental Table 2).

Among the technical challenges occurring during this study (discussed in ref. 21), the ability of mice to stand on the food hopper theoretically permitted grazing animals to consume up to their own BW in each feeding episode before anything was registered by the system. To overcome this potential drawback and permit the study of younger mice, the diameter of the food access aperture was reduced (Supplemental Figure 1).

### Study 4: The growth effects of meal-feeding and grazing are abolished in GHSR-null mice.

**Figure 3:**
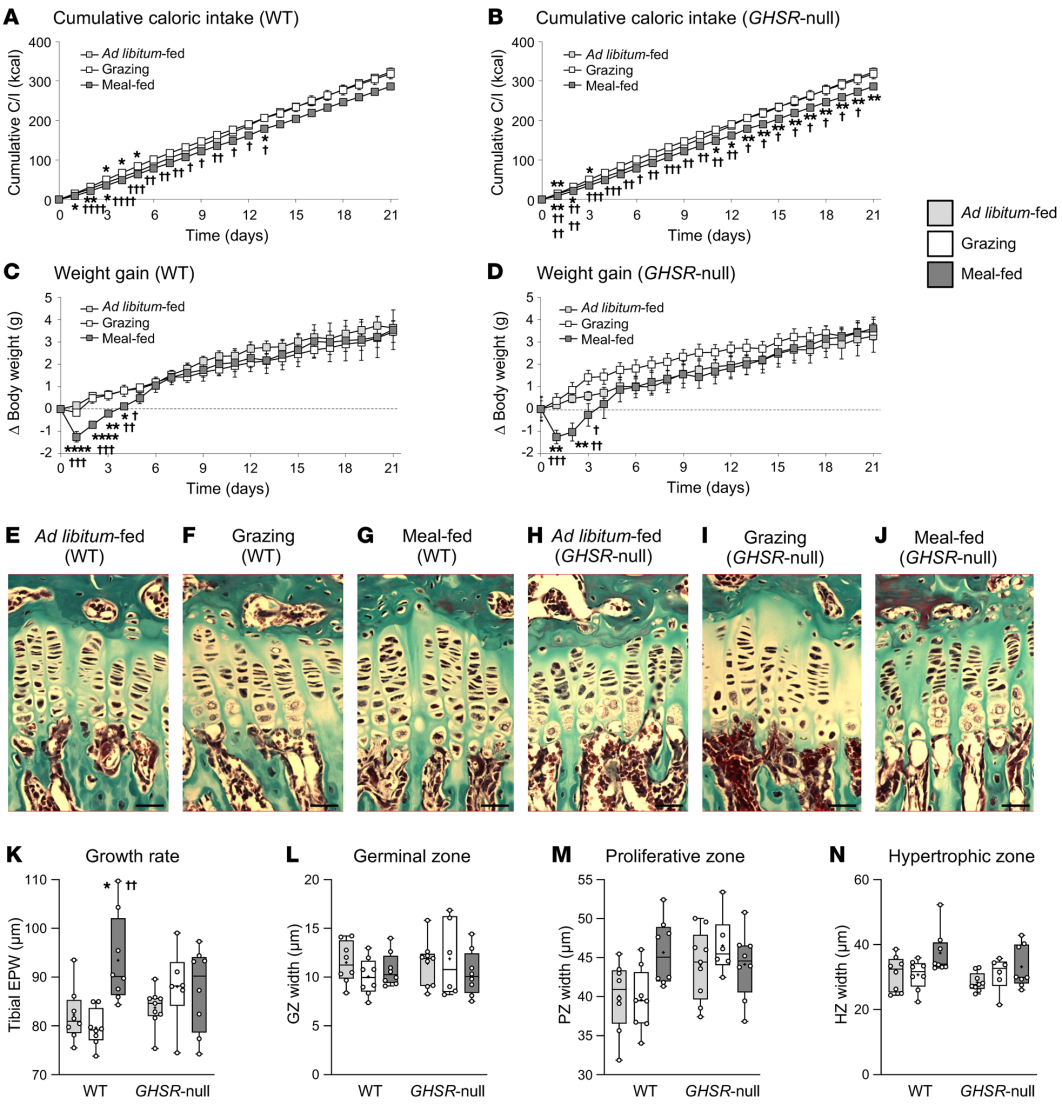
Meal-feeding promotes skeletal growth via a *GHSR*-dependent mechanism. Cumulative C/I (**A** and **B**), BW gain (**C** and **D**), tibia EPW (**K**), and germinal zone (**L**), proliferative zone (**M**), and hypertrophic zone (**N**) widths (measured in Masson’s trichrome–stained sections in **E**–**J**; scale bars: 20 μm) in 6-week-old male *GHSR*-null mice (**B**, **D**, and **H**–**J**) and their WT male littermates (**A**, **C**, and **E**–**G**) fed a standard, nonpurified rodent diet (13.9% AFE fat) in either ad libitum– (light gray symbols/bars), grazing (white symbols/bars), or meal-feeding (dark gray symbols/bars) patterns for 3 weeks. Data shown are the mean ± SEM (**A**–**D**), with box-and-whisker plots (**K**–**N**) showing the median line, mean (+), upper and lower quartile range (bars), data range (whiskers), and individual data points (*n* = 9, ad libitum–fed *GHSR*-null; *n* = 8, all other groups). Statistical comparisons were performed by 1-way ANOVA and Bonferroni’s selected-pairs post hoc test. *P < 0.05, **P < 0.01, and ****P < 0.0001 versus ad libitum–fed males (same genotype); ^†^*P* < 0.05; ^††^*P* < 0.01, ^†††^*P* < 0.001, and ^††††^*P* < 0.0001 versus grazing males (same genotype).

Deletion of *ghrelin* removes ghrelin, des-acyl ghrelin, and obestatin, while leaving the action of liver-enriched antimicrobial peptide 2 (LEAP2) intact. To delineate the role of this system further, we characterized the effect of these feeding patterns in juvenile mice in which transcription of the receptor for ghrelin, GHSR, was blocked. With a more tightly controlled grazing allowance, younger WT mice showed only transient hyperphagia (Figure 3A), with no effect on weight gain (Figure 3C). In contrast, the meal-fed younger mice displayed a transient reduction in caloric intake on days 1–3 (vs. ad libitum–fed mice) (Figure 3A), with BW gain only significantly reduced on days 2–4 (Figure 3C). These effects on caloric intake and weight gain were largely replicated in *GHSR*-null animals (Figure 3, B and D), with the exception that final cumulative caloric intake was reduced by 15% in meal-fed *GHSR*-null mice (*P* = 0.0036 vs. ad libitum–fed; *P* = 0.057 vs. grazing). Neither feeding pattern affected body length, tibia length, or organ size (Supplemental Table 3). However, although the skeletal growth rate was unaffected in grazing WT males (Figure 3, F and K), meal-feeding elevated the tibia EPW by 14% (*P* = 0.0166 vs. ad libitum–fed, *P* = 0.0009 vs. grazing; Figure 3, E, G and K), with the mean proliferative zone width and mean hypertrophic zone width in meal-fed WT mice being 116% (*P* = 0.1051; Figure 3M) and 117% (*P* = 0.2281; Figure 3N), respectively, of that in ad libitum–fed mice. These growth rate effects were entirely abolished in the absence of the GHSR (Figure 3, H–N). Although not significantly different, the profile of IGF-1 concentrations was broadly similar to the growth rate (Supplemental Table 3).

### Study 5: Meal-feeding and grazing produce different circulating ghrelin profiles.

Since these growth-promoting effects of grazing and meal-feeding are ghrelin/GHSR dependent, we characterized the effect of these feeding patterns on the dynamics of ghrelin secretion in chronically catheterized pattern-fed rats. Catheterization did not alter the effect of these feeding patterns on caloric intake, which remained similar to that observed in study 1 (cumulative caloric intake was reduced by 15% [*P* = 0.0001] and 12% [*P* = 0.0008] in grazing and meal-fed rats, respectively; Supplemental Table 4).

**Figure 4:**
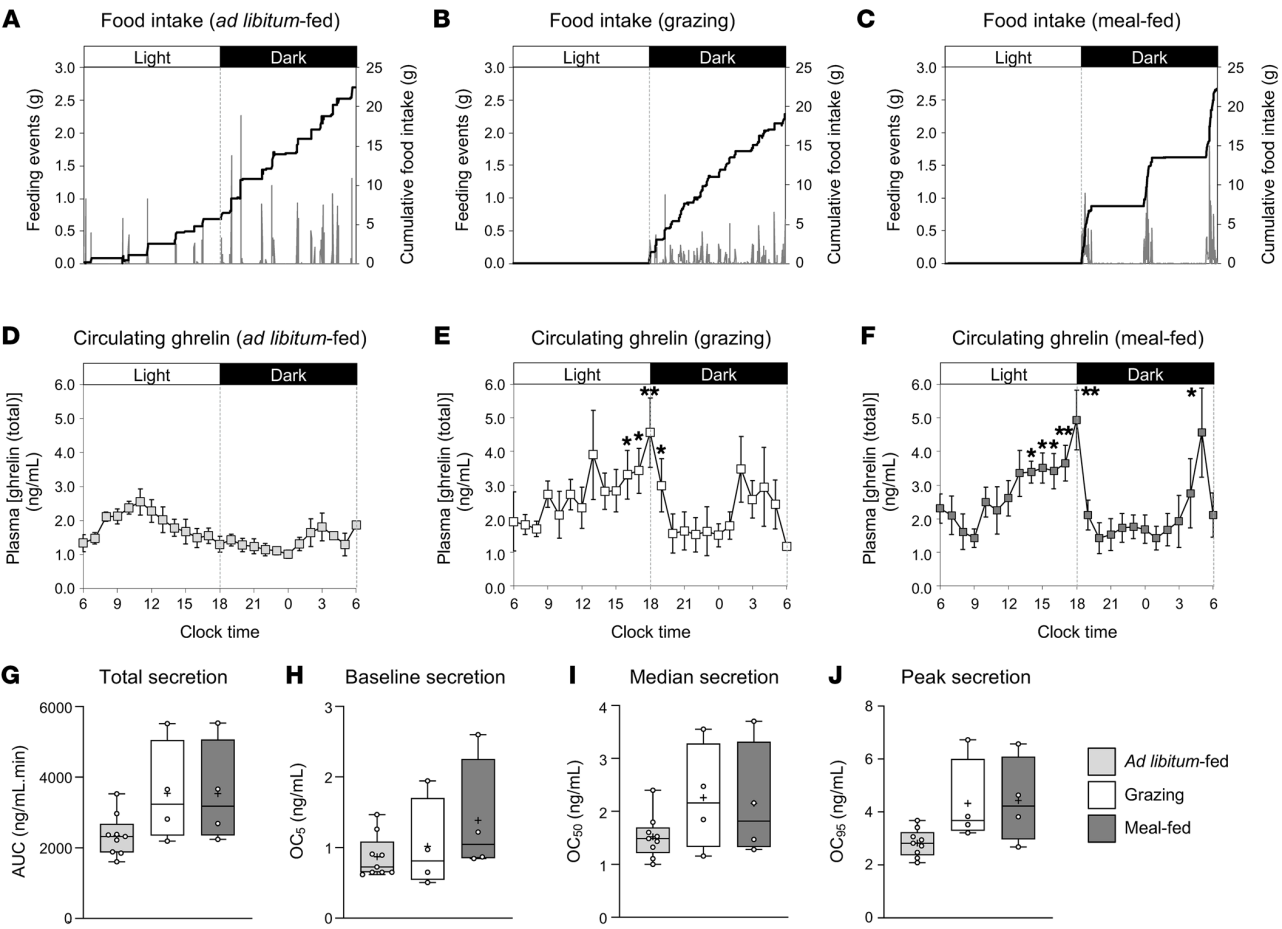
Grazing and meal-feeding modify circulating ghrelin profiles. Mean food intake profiles (**A**–**C**) and circulating ghrelin (total) profiles (**D**–**F**) in male rats fed a standard, nonpurified rodent diet in either ad libitum (**A** and **D**), grazing (**B** and **E**), or meal-feeding (**C** and **F**) patterns. Food intake profiles show individual feeding events (vertical gray bars) and cumulative intake (solid black line). Total ghrelin secretion (AUC) (**G**), baseline secretion (observed concentration at 5% [OC_5_]) (**H**), median secretion (OC at 50% [OC_50_]) (**I**), and peak ghrelin secretion (OC at 95% [OC_95_]) (**J**) are also shown. Ghrelin data shown are the mean ± SEM (**D**–**F**); box-and-whisker plots (**G**–**J**) show the median line, mean (+), upper and lower quartile range (bars), data range (whiskers), and individual data points (*n* = 9, ad libitum; *n* = 4, grazing and meal-fed rats), with statistical comparisons performed by 1-way ANOVA and Bonferroni’s selected-pairs post hoc test. Rat study: *P < 0.05 and **P < 0.01 versus ad libitum–fed.

Although total ghrelin secretion (AUC) in grazing and meal-fed rats was 150% of that in ad libitum–fed animals (Figure 4G), these means were not significantly different (*P* = 0.238 and *P* = 0.246 vs. ad libitum–fed, respectively). Mean peak ghrelin levels in grazing and meal-fed rats were 153% and 148%, respectively, of that in ad libitum–fed rats (*P* = 0.101 and *P* = 0.154, respectively; Figure 4J), with neither baseline (Figure 4H) nor median (Figure 4I) secretion being significantly different. Circulating ghrelin levels in ad libitum–fed rats showed the expected circadian rhythm (30), with the peak concentration occurring at 1100 hours (Figure 4D), immediately prior to the first major spontaneous feeding event (Figure 4A). Thereafter, circulating ghrelin declined, reaching a nadir at 2400/0000 hours (Figure 4D). In contrast, plasma ghrelin concentrations increased across the light phase in grazing (Figure 4E) and meal-fed (Figure 4F) rats prior to the commencement of nocturnal feeding (Figure 4, B and C). Although commencement of feeding produced a precipitous decline in circulating ghrelin levels in grazing and meal-fed rats (Figure 4, E and F), ghrelin levels remained higher in grazing rats at 1900 hours (Figure 4E). Despite constant food intake throughout the dark phase (Figure 4B), grazing was accompanied by a sustained doubling in mean circulating ghrelin in the second half of the dark phase (from 0200 to 0500 hours; *P* = 0.09 vs. ad libitum–fed; Figure 4E). Although meal-fed rats failed to show a preprandial rise in ghrelin before the second (midnight) meal, a trebling of circulating ghrelin levels occurred between 0100 hours and 0500 hours prior to the end-dark phase meal, declining sharply with the commencement of feeding (Figure 4, C and F). Thus, while grazing failed to maintain suppressed circulating ghrelin levels, meal-feeding produced a rapid reduction in ghrelin secretion.

### Study 6: Meal-feeding enhances GH pulsatility in rats.

**Figure 5:**
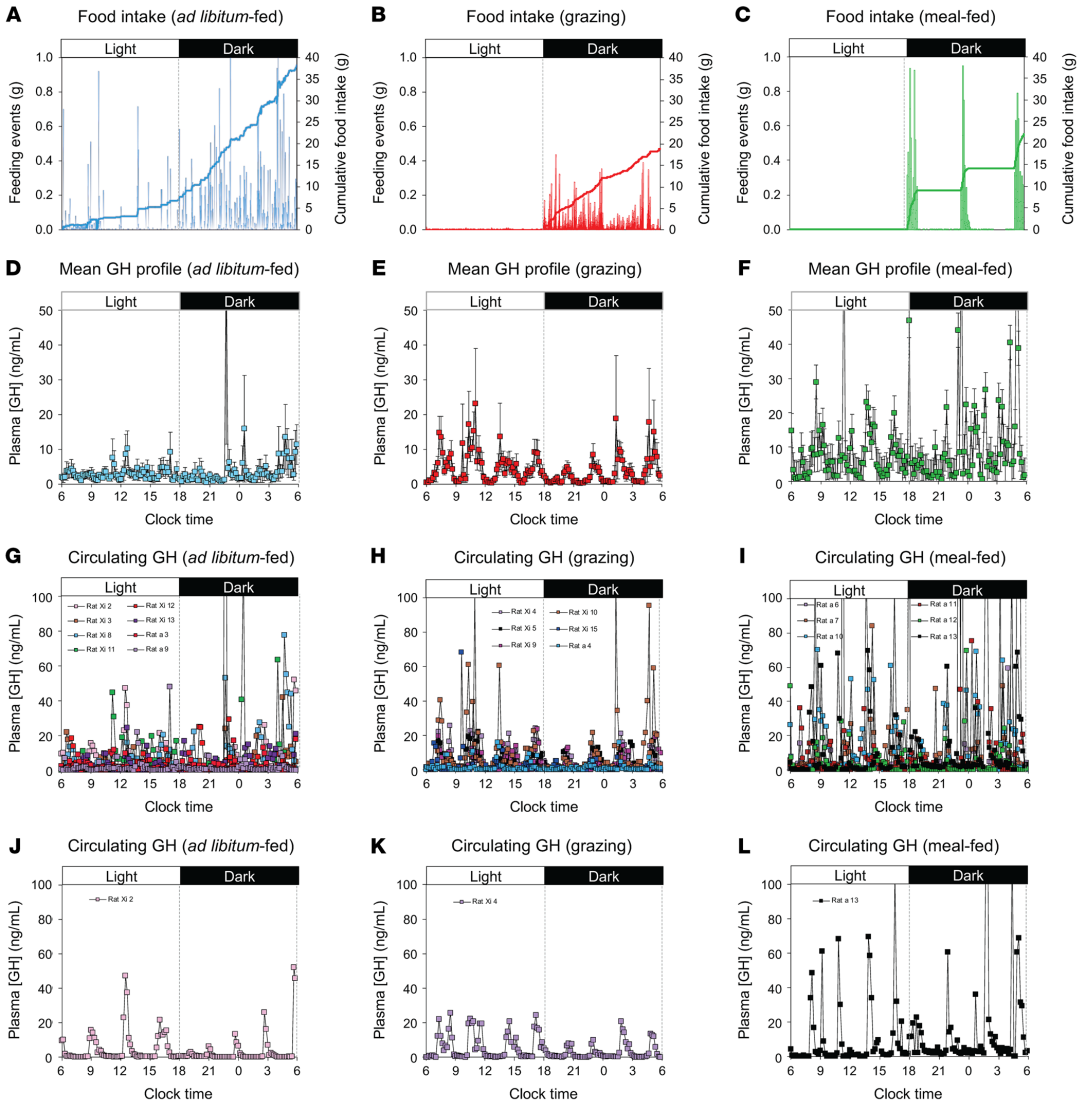
Meal-feeding amplifies GH rhythmicity. Mean food intake profiles (**A**–**C**), mean GH profiles (± SEM) (**D**–**F**), superimposed individual GH profiles (**G**–**I**), and representative individual GH profiles (**J**–**L**) in male rats fed a standard, nonpurified rodent diet in either ad libitum (**A**, **D**, **G**, and **J**) (*n* = 8), grazing (**B**, **E**, **H**, and **K**) (*n* = 6), or meal-feeding (**C**, **F**, **I**, and **L**) (*n* = 6) patterns. Food intake profiles (**A**–**C**) show individual feeding events (vertical bars) and cumulative intake (solid line).

**Figure 6:**
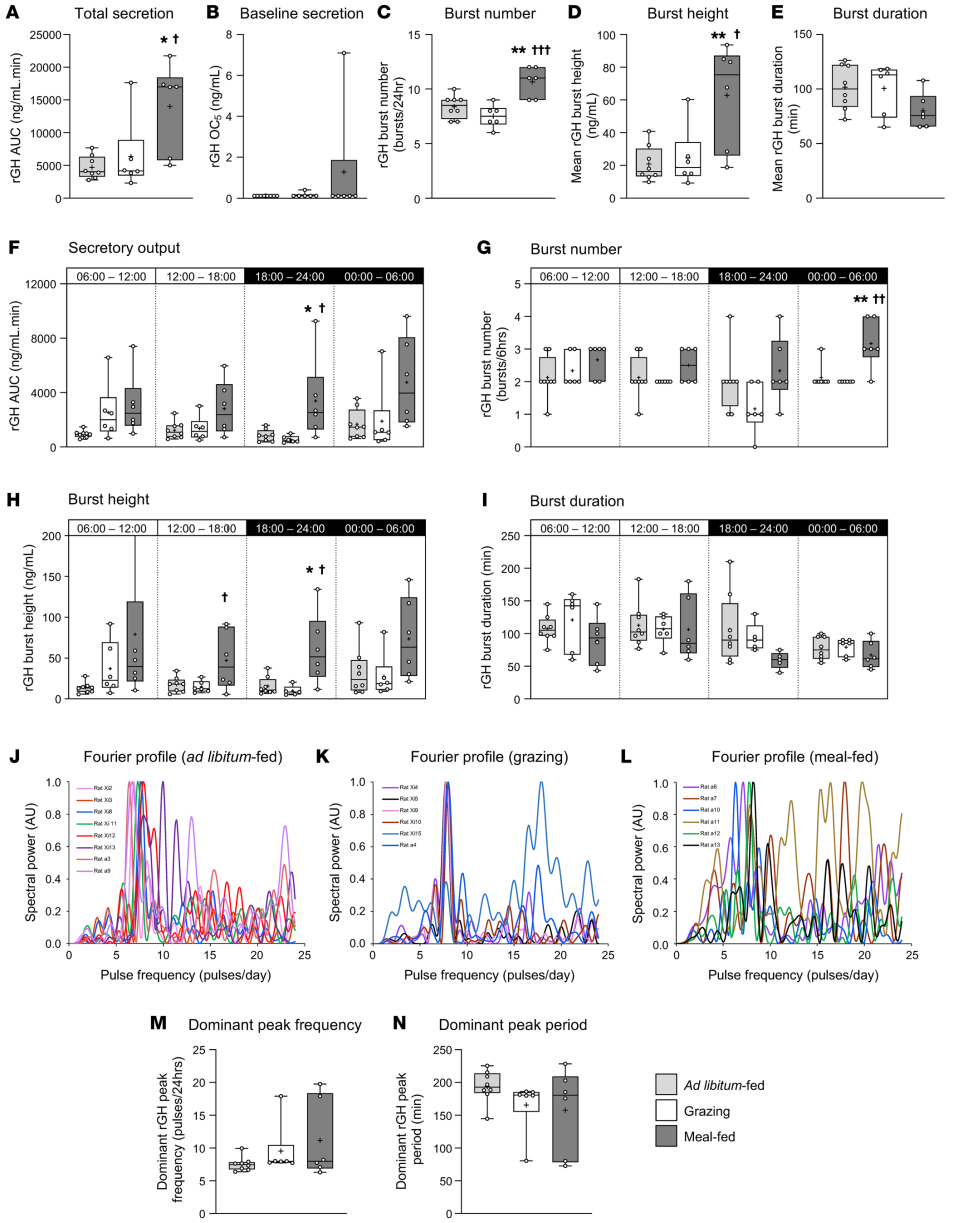
Meal-feeding enhances GH pulsatility predominantly in the dark phase. GH secretory output (AUC) (**A** and **F**), baseline secretion (OC_5_) (**B**), burst number (**C** and **G**), burst height (**D** and **H**), and burst duration (**E** and **I**) for the full 24-hour period (**A**–**E**) or subdivided into the 6-hour periods representing the first or second half of the light and dark phases (**F**–**I**) in male rats fed a standard, nonpurified rodent diet in either ad libitum (**A**) (*n* = 8), grazing (**B**) (*n* = 6), or meal-feeding (**C**) (*n* = 6) patterns. Fourier analysis of GH frequency spectra (normalized spectral power in AU) (**J**–**L**) enabled derivation of dominant GH peak frequency (**M**) and period (**N**). Data shown are individual spectral power profiles (**J**–**L**), box-and-whisker plots (**A**–**I**, **M**, and **N**) show the median line, mean (+), upper and lower quartile range (bars), data range (whiskers), and individual data points, with statistical comparisons performed by 1-way ANOVA and Bonferroni’s post hoc test. *P < 0.05 and **P < 0.01 vs. ad libitum–fed; ^†^*P* < 0.05, ^††^*P* < 0.01, and ^†††^*P* < 0.001 vs. grazing.

Given that ghrelin promotes GH secretion in a pattern-dependent manner (28), we characterized GH secretory dynamics in grazing and meal-fed rats. Ad libitum–fed animals (Figure 5A) showed episodic GH secretion characteristic of male rats (31, 32), with 8–9 bursts of GH occurring during each 24-hour period, separated by troughs in which GH was virtually undetectable (Figure 5, D, G, and J, and Supplemental Figure 2A). These bursts of GH secretion were unsynchronized between individual animals (Figure 5, D and G). Despite showing a reduction in cumulative caloric intake similar to that reported in studies 1 and 2 (Figure 5B and Supplemental Table 5), grazing had no effect on total (Figure 6A) or baseline (observed concentration 5 [OC5]) (Figure 6B) GH secretion, or the parameters of secretory dynamics (Figure 6, C–N), but induced inter-animal burst synchronization (Figure 5, E and H, and Supplemental Figure 2B). In contrast, despite inducing the same reduction in caloric intake (Figure 5C and Supplemental Table 5), meal-feeding almost tripled total GH secretion (*P* = 0.013 vs. ad libitum–fed, *P* = 0.047 vs. grazing; Figure 5, F, I, and L, Supplemental Figure 2C, and Figure 6A), inducing a degree of synchronization (Figure 5F) without significantly influencing baseline secretion (Figure 6B). Fourier analysis revealed that, while the dominant period in all 3 feeding patterns remained in the 150- to 200-minute range (7.2–9.6 bursts per day; Figure 6, J–N), meal-feeding was accompanied by the presence of numerous peaks in the higher frequency range (Figure 6L), without influencing the dominant period or frequency significantly (Figure 6, M and N). A simple “burst” metric revealed that meal-feeding elicited 2 additional secretory bursts per day (*P* = 0.0057 vs. ad libitum–fed, *P* = 0.0006 vs. grazing; Figure 6C), which coincided with the second preprandial ghrelin surge in the second half of the dark phase (Figure 6G). A tripling of mean burst height (*P* = 0.0054 vs. ad libitum–fed, *P* = 0.0176 vs. grazing; Figure 6D) was most prominent in the second half of the light phase and first half of the dark phase (Figure 6H). Given that the mean burst duration in meal-fed rats was 79% of that in ad libitum–fed animals (*P* = 0.1868 vs. ad libitum–fed; Figure 6E), the burst mass was not significantly increased (*P* = 0.3496 vs. ad libitum–fed; data not shown). Thus, meal-feeding in rats was accompanied by an increase in the frequency and magnitude of spontaneous GH secretory bursts.

### Study 7: Meal-feeding enhances ghrelin and GH pulsatility in humans.

**Figure 7:**
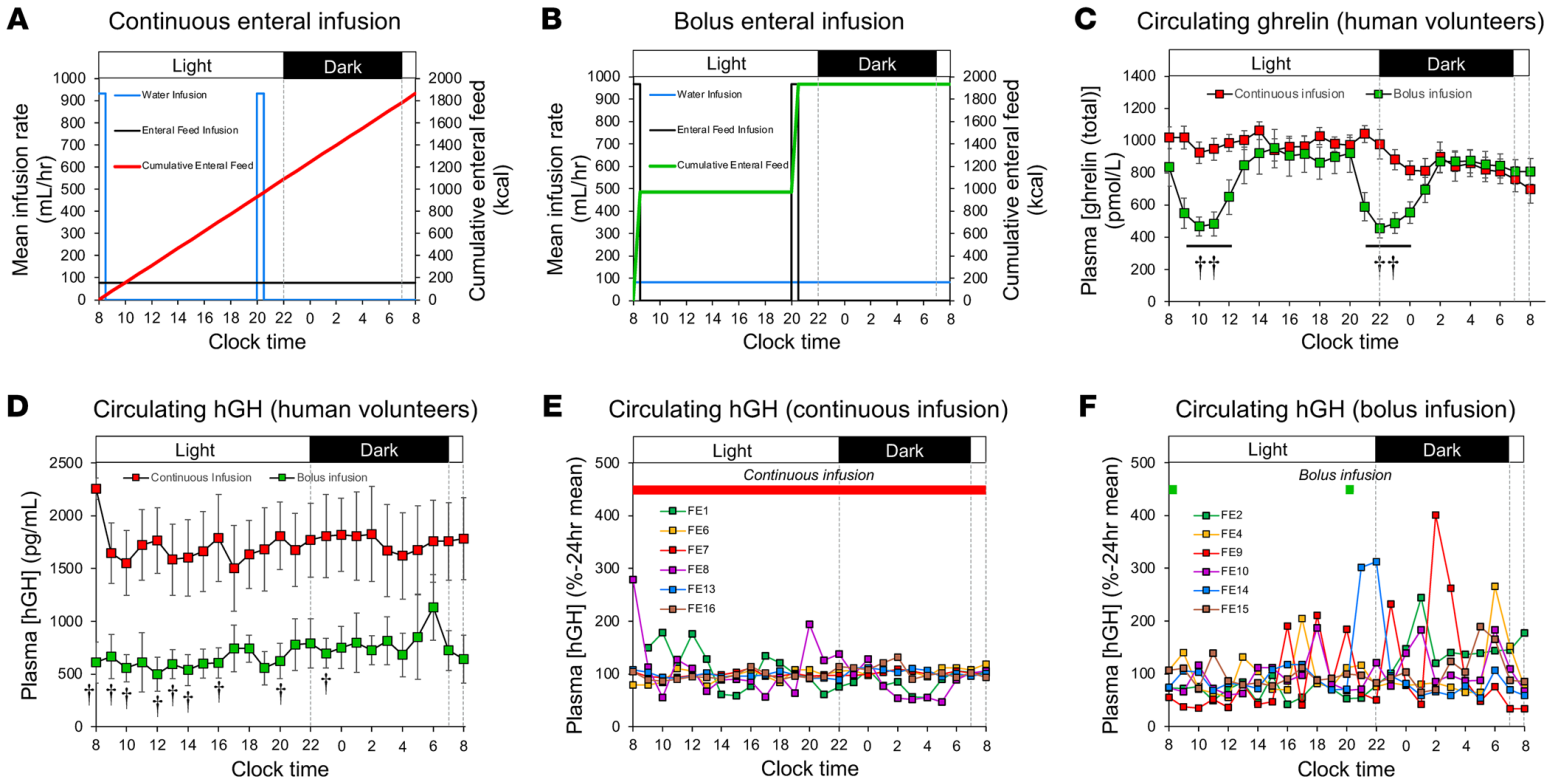
Meal-feeding enhances ghrelin and GH rhythmicity in human males. Human male volunteers received infusions of water (blue lines) and enteral feed via a nasogastric tube, with enteral feed given as a continuous (grazing, red line) (**A**) or bolus (meal-fed, green line) (**B**) infusion and water administered in the opposite profile. Circulating ghrelin (**C**) and hGH (**D**) data presented are the mean ± SEM (*n* = 8, both infusion patterns), with statistical comparisons performed by 1-tailed unpaired Student’s *t* test. ^†^*P* < 0.05 and ^††^*P* < 0.01 versus continuous infusion. In addition, individual circulating hGH profiles normalized (to each individual profile mean) in continuously infused (**E**) and bolus-infused (**F**) human male volunteers are presented.

To determine whether these feeding pattern–induced changes in the dynamics of ghrelin and GH secretion are replicated in humans, healthy male volunteers received enteral liquid formula through a nasogastric tube in either two 30-minute bolus infusions (at 0800 hours and 2000 hours; Figure 7B) or an equicaloric continuous infusion for 24 hours (Figure 7A). Analysis of hourly blood samples revealed that bolus-infused volunteers displayed a 4-hour suppression of circulating ghrelin levels after each infusion (Figure 7C). In contrast, circulating ghrelin remained at preprandial levels in continuously infused volunteers (Figure 7C). In addition, continuous nasogastric infusion produced consistently high circulating human GH (hGH) levels (Figure 7D). Since the lower sampling frequency did not permit rigorous pulse analysis, normalizing the values to the 24-hour profile mean for each individual revealed that volunteers who underwent continuous infusion had minimal ultradian rhythmicity (Figure 7E). In contrast, a postprandial fall in hGH in bolus-infused volunteers (60% lower after the first bolus than in continuously infused participants; *P* < 0.05; Figure 7D) was followed by the emergence of marked individual ultradian rhythmicity in all 6 individuals (Figure 7F). This was especially prominent following the second bolus infusion.

Thus, while grazing was insufficient to maintain postprandial suppression of ghrelin secretion and was accompanied by elevated hGH exposure, meal-feeding induced intermittent ghrelin exposure and enhanced hGH pulsatility.

## Discussion

Direct mechanistic evidence that ultradian feeding patterns influence physiological outcomes has been lacking. To address this deficit, we have exploited the flexibility and reliability of the CLAMS system to determine the effect of grazing and 3 meals a night on the endocrine regulation of growth in laboratory rodents. When combined with our evidence of parallel acute responses in humans, we believe our study presents the first direct evidence that temporal feeding patterns regulate indices of hormone secretory dynamics to influence developmental endpoints.

It is clear from our rodent studies that grazing slowed the rate of longitudinal growth in the tibial epiphyseal plate. We initially assumed from study 1 that this was due to the noticeable reduction in caloric intake, but when this phenomenon was repeated in older mice in the context of maintained, or even partially elevated, food intake (study 3), it was clear that nutritional restriction was not the underlying cause. However, the abolition of the grazing-induced reduction in growth rate in *ghrelin*-KO mice clearly implies a contribution for this gastric hormone, or potentially one of its coproducts. Our analysis of ghrelin profiles indicates that nocturnal grazing magnified the amplitude of the daily ghrelin rhythm seen in ad libitum–fed rats, with the addition of a large anticipatory surge (27) before the commencement of dark-phase feeding. This pattern of ghrelin exposure was insufficient to reduce total GH output in rats or alter the indices of GH burst dynamics that determine its biological effectiveness (28, 29). The observed alignment of the GH bursts between individual rats was intriguing and deserves comment. The commencement of the light phase is a powerful entraining signal for the GH axis (33), but drift in individual periodicity enables progressive misalignment between individuals. The large daily preprandial ghrelin surge immediately prior to the commencement of the dark phase (i.e., in 3-hour phase with the lights-on entrainment) acts as an additional entraining signal at the obverse side of the light-dark cycle to reinforce GH burst alignment.

It remains unclear at present how these changes in ghrelin secretion could influence skeletal growth in the absence of altered GH secretion. One possibility is a direct action of ghrelin in the growth plate. It has been reported that ghrelin (34), the GHSR (35, 36), and the activating enzyme ghrelin *O*-acyl transferase (37, 38) are expressed in chondrocytes, especially in the proliferative and hypertrophic zones where the effects of grazing are most prominent (Figure 1), but whether expression of these components is modified by feeding patterns remains to be determined. A potential paracrine or autocrine stimulation of chondrocyte GHSR was supported by the 7% reduction in BW observed in *ghrelin*-KO mice (*P* = 0.0003; data not shown) at the start of the study.

Our human study indicates an additional mechanism. Slow continuous nasogastric infusion of enteral feed for 24 hours failed to suppress circulating ghrelin, which remained at preprandial levels throughout the feeding period. In the short term, this was accompanied by a sustained elevation in circulating hGH levels. The difference between this result and our rat study is likely to reflect the period of feeding (24-hour infusion in humans vs. 12 hours of grazing for rats) and the shorter duration of the human study. Indeed, we have previously shown in rats that a week-long continuous infusion of ghrelin or a GHSR agonist reduces skeletal growth (29) by suppressing GH secretion (28). Thus, the sustained starvation signal that is represented by continuously elevated ghrelin is most likely to result in reduced GH secretion and impaired growth outcomes in the long term, even in the context of maintained nutrient supply.

In contrast to grazing, nocturnal meal-feeding defends skeletal growth in the context of caloric restriction, even accelerating the growth rate in younger mice. To see this effect reflected in measurable changes in tibia length will likely require longer studies, but the lack of meal-induced growth rate enhancement in the absence of GHSR expression and the reversal of the effect in *ghrelin*-KO mice clearly imply a role for the acylated form of ghrelin. At first glance, however, there appears to be little difference in the circulating ghrelin profiles between grazing and meal-fed rats, with overall, median, and peak secretion being entirely comparable. This serves to emphasize the importance of timing in eliciting the observed effects, with meal-fed rats displaying transient preprandial peaks before the first and third meals. Our evidence that a twice-daily bolus nasogastric infusion of enteral feed elicited matched suppressions of ghrelin secretion in humans not only concurs with early evidence of preprandial surges of ghrelin in humans (26), but confirms that meal-feeding results in intermittent ghrelin exposure.

While we cannot exclude the possibility of a direct action of ghrelin in the growth plate, the enlargement of the proliferative and hypertrophic zones and the increased chondrocyte migration rate imply augmentation of GH/IGF-1 axis activity. Thus, the trebling of GH secretion in rats, resulting from a combination of doubled pulse height and increased burst frequency, appears to be the most likely mechanism. We have reported a similar effect on GH pulse height and skeletal growth in response to intermittent intravenous infusion of ghrelin (28, 29), but the change in burst frequency is more unusual. While meal-fed rodents were subjected to the same triggering influences of the dark/light interface and the large pre-dark phase surge in ghrelin as grazing animals, the meals commencing at 2330 hours and 0500 hours, the latter with an accompanying pre-prandial ghrelin surge, represent two additional temporal cues. Since these were not separated by multiples of 3 hours, but by multiples of 2.75 hours, this appears to have had a “concertinaring” effect, shortening the refractory period between individual GH bursts, thereby permitting 2 additional bursts per day. Spontaneous bursts of GH secretion in male rodents are thought to occur when peaks of GH-releasing hormone (GHRH) secretion coincide with a trough in somatostatin secretion (39). The lack of a shift in the period of the peak frequency in the Fourier profiles (Figure 6) suggests that the mechanism giving rise to this dominant frequency was largely unaffected by these feeding patterns. However, the emergence of additional bursts in meal-fed rats suggests additional somatostatin troughs, especially in the dark phase, while the elevation in burst height was most likely due to larger GHRH bursts.

At first glance, these findings do not appear to be replicated in our human data, as acute bolus nasogastric infusions were accompanied by lower overall circulating GH compared with “grazing” humans. However, the growth-promoting action of GH is not determined solely by the level of exposure or total exposure time, as pulsed infusions of GH are more effective in promoting growth in rats (40) and elevating bone formation markers in humans (41). In this context, the emergence of pulsatile GH secretion in all 6 bolus-infused volunteers is significant and corroborates evidence that prominent GH pulsatility emerges in male volunteers after midnight (42). Taken together, our data indicate that meal-feeding augmented GH pulsatility, increasing the number of GH bursts in rats to the optimal range for promoting axial growth.

We believe our data have a number of important implications. From a narrow perspective, our human study indicates that, in addition to content and the total delivery rate (43), the physiological effectiveness of enteral feeding was determined by the effect of the delivery pattern on hormone profiles. Second, while our study focused on the effect of feeding patterns on the growth axis, it is clear that the effect of ghrelin and GH on a wide range of physiological endpoints, including the regulation of fat mass, insulin sensitivity, epigenetic mechanisms, and drug metabolism is pattern dependent (44–46) and therefore potentially susceptible to changes in feeding pattern. Indeed, it is possible that the effect of manipulating feeding patterns to enhance GH pulsatility in females will be more dramatic. Taken together, our data imply that the contemporary shift from regular meals to snacking behavior (47, 48) may be detrimental to optimal skeletal growth outcomes, particularly in the context of undernourishment.

## Methods

### Sex as a biological variable

Our study examined the effects in male rodents and humans because the GH secretory profile is more amenable to the quantification of changes in the variables of pulsatility. It is unclear whether the findings we report will be applicable in females.

### Animals

Male Sprague-Dawley rats (studies 1, 2, 5, and 6) were purchased from Charles River Laboratories and housed upon receipt as described below. Male WT mice (C57/Bl6J) and their homozygous *ghrelin*-KO (study 3) and *GHSR*-null (loxTB-GHSR, study 4) littermates were obtained from heterozygous × heterozygous matings of breeding stock derived from embryos (*ghrelin*-KO) or mice (*GHSR*-null) imported from the vivaria at Baylor College of Medicine (Houston, Texas, USA) and the University of Texas Southwestern (Dallas, Texas, USA) respectively. Genotype identification was performed by PCR analysis of DNA extracted from ear punches, as previously described (23, 24).

All experimental animals were individually housed in the metabolic room of the BIOSV Animal Facility at Cardiff University, under 12-hour light/12-hour dark (lights on at 0600 hours) conditions, with water available ad libitum and diet supplied in 1 of 3 patterns as previously described (21) and summarized briefly below.

#### Nocturnal grazing.

Grazing animals were permitted to eat 1/24 of the mean total daily food consumption of a concurrent cohort of 3 age-matched, ad libitum–fed control animals every 30 minutes during the dark phase, with the first access period coinciding with lights out (1800 hours). This allowance increased in parallel with the daily food intake of the growing ad libitum–fed control animals. Thus, grazing rats were denied large meals.

#### Nocturnal meal-feeding.

Meal-fed animals were permitted three 1-hour periods of ad libitum dietary access at the beginning (1800 hours), middle (2330 hours), and end (0500 hours) of the dark phase, with the access lid remaining closed at all other times. Thus, meal-fed animals were not permitted to graze between meals.

#### Ad libitum feeding.

In order to calculate the food intake allowance for grazing animals, cohorts of age- and weight-matched animals were housed in either standard transparent cages (rats, catalog 2154; Tecniplast UK Ltd.) or metabolic cages (mice, catalog 3700M061, Tecniplast UK Ltd.) and permitted ad libitum access to the same crushed diet (see dietary information below). Food consumption was quantified daily between 0900 and 1000 hours. The effectiveness of this approach and a more detailed description of procedural considerations have been published previously (21).

### Human volunteers

Sixteen healthy male volunteers (study 7; Supplemental Table 6) were recruited via local advertisement. General health and validated chronotype questionnaires were used to screen participants and assess habitual sleep patterns and diurnal preferences (49–51).

### Study 1: Meal-feeding protects skeletal growth in male rats

Three groups of 4-week-old male Sprague-Dawley rats (weighing 83.8–118.8 g) were fed standard, nonpurified rodent chow (SRC, SDS RM3, Special Diet Services Ltd.) containing 4.2% crude fat (AFE 13.9% fat), 22.4% crude protein, 4.2% crude fiber, and 7.6% crude ash (see ref. 21 for full dietary components) in either ad libitum, grazing, or meal-fed patterns for 6 weeks. Food intake and BW were quantified daily. After weighing on day 42, each rat was anesthetized with isoflurane, the nose-anus length was measured, and the rat was decapitated. The right tibia was dissected and its length measured with a hand-held micrometer. Tibiae were fixed in buffered formal saline for 48 hours at 4°C and decalcified in 0.5 M EDTA (pH7.6) for more than 3 weeks, before being stored in 70% ethanol at 4°C for subsequent quantification of the EPW. Two animals were omitted from the ad libitum–fed group, 1 that had cumulative food intake greater than 2 times the SD from the mean, and 1 with a BW gain greater than 2 times the SD from the mean.

### Study 2: Grazing reduces the rate of chondrocyte migration

Three groups of 4-week-old male Sprague-Dawley rats (weighing 81.3–127.5 g) received BrdU (1 mg/kg; i.p.) on 3 consecutive days and were fed SRC ad libitum. After 5 days, the rats continued to receive SRC in either ad libitum, grazing, or meal-fed patterns for 3 weeks. At the end of this period, the rats were anesthetized (Dolethal 200 mg/kg, i.p.; Vetoquinol UK Ltd.) and killed by transcardial perfusion fixation. Tibiae were excised, measured for length, and processed as described above for quantification of total EPW and zonal widths and the migration of BrdU^+^ cells by IHC (see below). One animal was omitted from the grazing and meal-fed groups as more than 2 variables differed from the mean by greater than 2 times the SD and another from the meal-fed group because the tibia EP was sheared.

### Study 3: The growth effects of meal-feeding and grazing are reversed in ghrelin-KO mice

Three groups of 6-month-old male *ghrelin*-KO mice (BW: 28.4–33.9 g; 30.9 ± 0.5 g, *n* = 18) and male WT littermates (BW: 29.4–37.4 g; 33.1 ± 0.5 g, *n* = 18; *P* < 0.01) were permitted to consume SRC in either ad libitum, grazing, or meal-fed patterns. Older mice were used because they were big enough to be housed in the unmodified rat CLAMS cages. Meal-feeding was done as described above, but grazing mice were permitted to consume 0.5 g (~11% of total ad libitum food intake) every 30 minutes during the dark phase throughout the study. After 3 weeks of exposure to these dietary patterns, during which BW and daily food consumption were monitored daily, mice were anesthetized with isoflurane and killed by decapitation. Plasma separated from trunk blood samples was stored at –80°C prior to quantification of circulating IGF-1 levels, with pituitary, liver, kidney, and adrenal glands dissected and weighed. Tibiae were collected as in study 1.

### Study 4: The growth effects of meal-feeding and grazing are abolished in GHSR-null mice

Three groups of 6-week-old male *GHSR*-null mice (BW: 18.18–20.72 g; 18.56 ± 0.28 g, *n* = 23) and 3 groups of male WT littermates (BW: 12.99–21.59 g; 19.25 ± 0.38 g, *n* = 24; *P* = 0.047) were permitted to consume SRC in either ad libitum, grazing, or meal-fed patterns. Meal-feeding was applied as above, but grazing mice were permitted to consume 0.2 g every 30 minutes during the dark phase throughout the study. After 3 weeks, mice were anesthetized with Dolethal (as above) and killed by decapitation, with tissues collected as in study 3.

### Study 5: Meal-feeding and grazing produce different circulating ghrelin profiles

Three groups of 4-week-old male Sprague-Dawley rats were fed SRC in either ad libitum, grazing, or meal-fed patterns for 3 weeks. On day 18, rats were anesthetized with isoflurane and prepared with a single-bore right jugular vein catheter, as previously described (21, 33). After recovery from surgery, the catheters were connected to an automated blood sampling system, and patency was maintained by an hourly flushing protocol in which blood was drawn to the top of the catheter and returned to the rat with the infusion of a 20 μL bolus of sterile heparinized saline (10 IU/mL). After a further 48 hours, automated serial blood sampling was commenced, in which 100 μL of 1:2 blood (50 μL blood in 50 μL heparinized saline) was collected every hour for 24 hours, beginning at 0600 hours. Blood samples were collected on a refrigerated fraction collector bed, vortexed, and centrifuged at 2,773*g* at 4°C for 5 minutes, before 100 μL of 1:2 plasma was removed and stored at –20°C for subsequent determination of circulating ghrelin levels by radioimmunoassay (RIA) (see below). On day 21, rats were reanesthetized, the nose-anus length was measured, and the rats were then decapitated, with tissue collection done as above.

### Study 6: Meal-feeding enhances GH pulsatility in rats

Three groups of 4-week-old male Sprague-Dawley rats were fed SRC in either ad libitum, grazing, or meal-fed patterns for 3 weeks. On day 18, the rats were prepared with a single-bore right jugular vein catheter, and patency was maintained as above. After 48 hours, automated serial blood sampling was commenced, in which 100 μL of 1:5 blood (20 μL blood in 80 μL heparinized saline) was collected every 10 minutes for 24 hours, beginning at 0600 hours. Blood samples were collected on a refrigerated fraction collector bed, vortexed, and centrifuged at 2,773*g* at 4°C for 5 minutes, before 20 μL of 1:5 plasma was removed and stored at –20°C for subsequent determination of circulating GH levels by RIA (see below).

### Study 7: Meal-feeding enhances ghrelin and GH pulsatility in humans

Sixteen male volunteers (18–42 years of age) fitted with a nasogastric tube received liquid feed (Nestlé Peptamen): 100 kcal, 7.6 g carbohydrate; 3.8 g fat; 9.2 g protein; vitamins and minerals/100 mL standardized to the individual resting metabolic rate (Supplemental Table 6) in either two 30-minute bolus infusions (0800 hours and 2000 hours; 1,875 ± 117 kcal/day) or as a continuous infusion for 24 hours (1,910 ± 218 kcal/day) (lights on [800 lux] at 0700 hours; lights off [0 lux] at 2200 hours). To negate the potential confounding influence of gastric filling, bolus-infused and continuously-infused volunteers received continuous (82 ± 10 mL/h for 24 hours) or bolus (two 30-minute infusions at 0800 hours and 2000 hours) nasogastric water infusions, respectively (Figure 7, A and B). Hourly blood samples (10 mL) were withdrawn manually from an indwelling median cubital vein catheter into tubes containing EDTA and immediately centrifuged at 3,466*g* at 4°C for 10 minutes. Separated plasma was aliquoted and stored at –80°C prior to quantification of circulating ghrelin and hGH by ELISA.

### Tissue processing

#### Quantification of tibial growth rates.

Tibiae were fixed in buffered formal saline for 48 hours and then decalcified (in 10% EDTA for 3 weeks) and embedded in paraffin wax, with 7 μm longitudinal anterior-posterior sections collected and stained with Masson’s trichrome. The epiphyseal plate and individual zonal widths were measured under light microscopy (mean of 3 measurements per section, 3 sections per bone) using Leica Q-win software (version 3). BrdU^+^ nuclei were visualized by IHC (primary antibody: rat anti-BrdU, MCA2060, Bio-Rad; secondary antibody: goat anti–rat IgG, ImmPRESS-AP MP-5404, Vector Laboratories). The distance between the closest BrdU^+^ nuclei in each column (to the germinal zone) and the top of the germinal zone (Figure 1, L–N) was divided by the number of days between the last injection and the day of termination to obtain an index of actual growth rate.

#### Hormone quantification.

In the absence of protease inhibitor use to protect the acyl side chain, we were only able to quantify total ghrelin in rat and human samples. Plasma ghrelin (total) concentrations in rat samples were determined by radioimmunoassay (Millipore RIA kit GHRT-89HK, MilliporeSigma) according to the manufacturer’s instructions (quality control [QC] values fell within the specified ranges; QC1 = 0.54 ng/mL [range, 0.39–0.91 ng/mL]; QC2 = 1.43 ng/mL [range, 0.95–1.97 ng/mL]; intra-assay variation [IAV], 3.97%; sensitivity 0.13 ng/mL). Ghrelin (total) concentrations in human plasma samples were quantified by ELISA (Invitrogen ghrelin human kit BMS2192, Thermo Fisher Scientific) according to the manufacturer’s instructions.

Circulating GH concentrations were determined in rat plasma samples by RIA, with the results expressed in terms of the reference preparation RP-2 (rGH) using reagents supplied by the National Institute of Diabetes and Digestive and Kidney Diseases (NIDDK), NIH, and I^125^-labeled rGH (IRC-105, Institute of Isotopes Co. Ltd.) (IAV 2.62%; sensitivity 0.12 ng/mL). GH concentrations in human plasma samples were quantified by ELISA (human growth hormone DuoSet DY1067, R&D Systems) according to the manufacturer’s instructions. Plasma IGF-1 concentrations were determined in rodent samples by ELISA (Mouse/Rat IGF-1 DuoSet DY791, R&D Systems) according to the manufacturer’s instructions.

### Statistics

Feeding profiles for individual animals are presented as individual feeding events with the superimposition of corresponding cumulative food intake data or corresponding hormone profiles. Total hormone secretory output was determined by calculating the AUC (using Microsoft Excel version 16.15 for Mac). Given the episodic nature of GH secretion, several approaches were taken to characterize the parameters of secretion. Distribution analysis was used to estimate baseline secretion (OC_5_, which is the cut off value below which 5% of the samples fall when ranked in ascending order of concentration) (21). Using the distribution analysis output, secretory “bursts” were identified in which the value exceeded OC_80_ but returned to OC_30_ before the next burst. Burst duration represented the period in which GH concentration in consecutive samples was greater than OC_30_. Values were determined for the total 24-hour period and for four 6-hour periods representing the first and second halves of the light and dark phases. To analyze burst frequency in the rat GH data, missing data points were linearly interpolated and the data detrended using the smoothness priors approach (SPA) with the smoothing parameter set at 300 (52). The power spectrum of the detrended data was then computed using the discrete Fourier transform (DFT) applied to a 24-hour period time window. The dominant frequency was taken as the frequency value corresponding to the maximum spectral power of the discrete transform, which was calculated using a quadratic interpolation. These approaches were not applicable to the human data due to the lower sampling frequency. To visualize ultradian hGH variation, individual values were normalized to the profile mean for each volunteer and expressed as the percentage-mean.

Apart from representative profiles, all data are presented as either the mean (± SEM) or in box-and-whisker plots (showing the median line, mean [+], upper and lower quartile range [bars], data range [whiskers], and individual data points). Comparisons were made by 1-way ANOVA and Bonferroni’s selected-pairs post hoc test (GraphPad Prism, version 7.0d for Mac OS X) or 1-tailed unpaired Student’s t-test (Microsoft Excel version 18.86 for Mac), as indicated in the figure and table legends, with a *P* values of less than 0.05 considered significantly significant.

### Study approval

All animal procedures (including those in genetically modified mice) were performed under the authority of the Animals (Scientific Procedures) Act, 1986 (UK) Animal Research: Reporting of in vivo Experiments (ARRIVE) guidelines and were specifically approved by the Cardiff University Animal Welfare Ethical Review Body (UK Home Office Project License PP3727126). Human volunteers were fully briefed on the study requirements prior to provision of written informed consent. Procedures were conducted in accordance with the latest version of the Declaration of Helsinki and authorized by the National Health Service (NHS) research ethics committee (reference: 18/SW/0176), and the trial is registered at ClinicalTrials.gov (NCT03906409).

### Data availability

Underlying data for this publication are accessible in the Supporting Data Values file.

## Author contributions

JAB and TW conceptualized the study. JAB and TW designed the methodology. TW validated the results. AKEH, HAS, AMC, LHMW, JJW, and TW conducted formal analysis. AKEH, RCB, TWT, BAW, ALH, KDT, SKRR, LHMW, and TW performed experiments. YS and JMZ provided resources. TW wrote the original draft of the manuscript. AKEH, HAS, JAB, JSD, YS, and TW reviewed and edited the manuscript. TW performed visualization. JAB, JSD, and TW supervised the study. TW was responsible for project administration. JSD and TW acquired funding for the work.

## Supplementary Material

Supplemental data

Supporting data values

## Version 1 04/01/2025

In-Press Preview

## Version 2 06/16/2025

Electronic publication
