# Supplementary material for: Meal-feeding promotes skeletal growth by ghrelin-dependent enhancement of growth hormone rhythmicity
Source: J Clin Invest. 2025 Apr 1;135(12):e189202. doi: 10.1172/JCI189202 (PMC12165789; doi:10.1172/JCI189202)
Supplement: Supplemental data [file jci-135-189202-s307.pdf]

Supplementary Table 1: *Post-mortem* analysis of growth in pattern-fed rats (Study 2)

	Ad libitum (n=12)	**Grazing** (n=7)	**Meal-fed** (n=6)
Cumulative caloric intake (kcal)	2243 ± 43	1834 ± 42 [aaaa]	1849 ± 25 [aaaa]
Body weight gain (g)	165.9 ± 7.0	137.2 ± 5.8 [a]	162.9 ± 5.0
Tibial length (mm)	39.1 ± 0.4	38.3 ± 0.4	38.1 ± 0.3
Epiphyseal plate width (µm)	217.8 ± 8.3	179.6 ± 3.3 [aa]	205.8 ± 4.1
Germinal zone width (µm)	8.4 ± 0.2	7.7 ± 0.3	8.4 ± 0.4
Proliferative zone width (µm)	119.9 ± 5.3	96.4 ± 2.1 [aa]	116.2 ± 5.6
Hypertrophic zone width (µm)	87.3 ± 4.2	71.8 ± 3.7 [a]	80.3 ± 3.3

[a] $P<0.05$; [aa] $P<0.01$; [aaaa] $P<0.0001$ vs *Ad libitum*

Supplementary Table 2: *Post-mortem* analysis of growth end points in pattern-fed 6-month old WT and *ghrelin*-KO mice (Study 3)

	**WT**			***Ghrelin*-KO**		
	Ad libitum (n=6)	**Grazing** (n=5)	**Meal-fed** (n=7)	***Ad libitum*** (n=7)	**Grazing** (n=5)	**Meal-fed** (n=6)
Nose-anus length (cm)	9.87 ±0.09	9.98 ±0.09	9.73 ±0.08	9.83 ±0.07	9.64 ±0.13	9.78 ±0.19
Tibial length (mm)	18.11 ±0.31	18.20 ±0.20	17.97 ±0.05	17.88 ±0.15	18.11 ±0.21	18.17 ±0.14
Plasma [IGF-1] (ng/ml)	4089 ±921	2881 ±779	1844 ±492	2799 ±988	2911 ±917	1917 ±450
Pituitary weight (mg)	1.28 ±0.24	1.40 ±0.11	1.46 ±.010	1.44 ±0.06	1.14 ±0.22	1.28 ±0.12
Liver weight (%-BW)	4.91 ±0.12	4.47 ±0.36	4.84 ±0.07	4.65 ±0.16	4.60 ±0.23	4.72 ±0.29
Kidney weight (%-BW)	0.67 ±0.02	0.67 ±0.03	0.64 ±0.03	0.69 ±0.02	0.69 ±0.03	0.64 ±0.02
Adrenal weight (mg)	3.45 ±0.90	3.04 ±0.51	2.35 ±0.26	3.31 ±1.06	3.80 ±0.92	3.08 ±0.54

Values shown are mean ± SEM

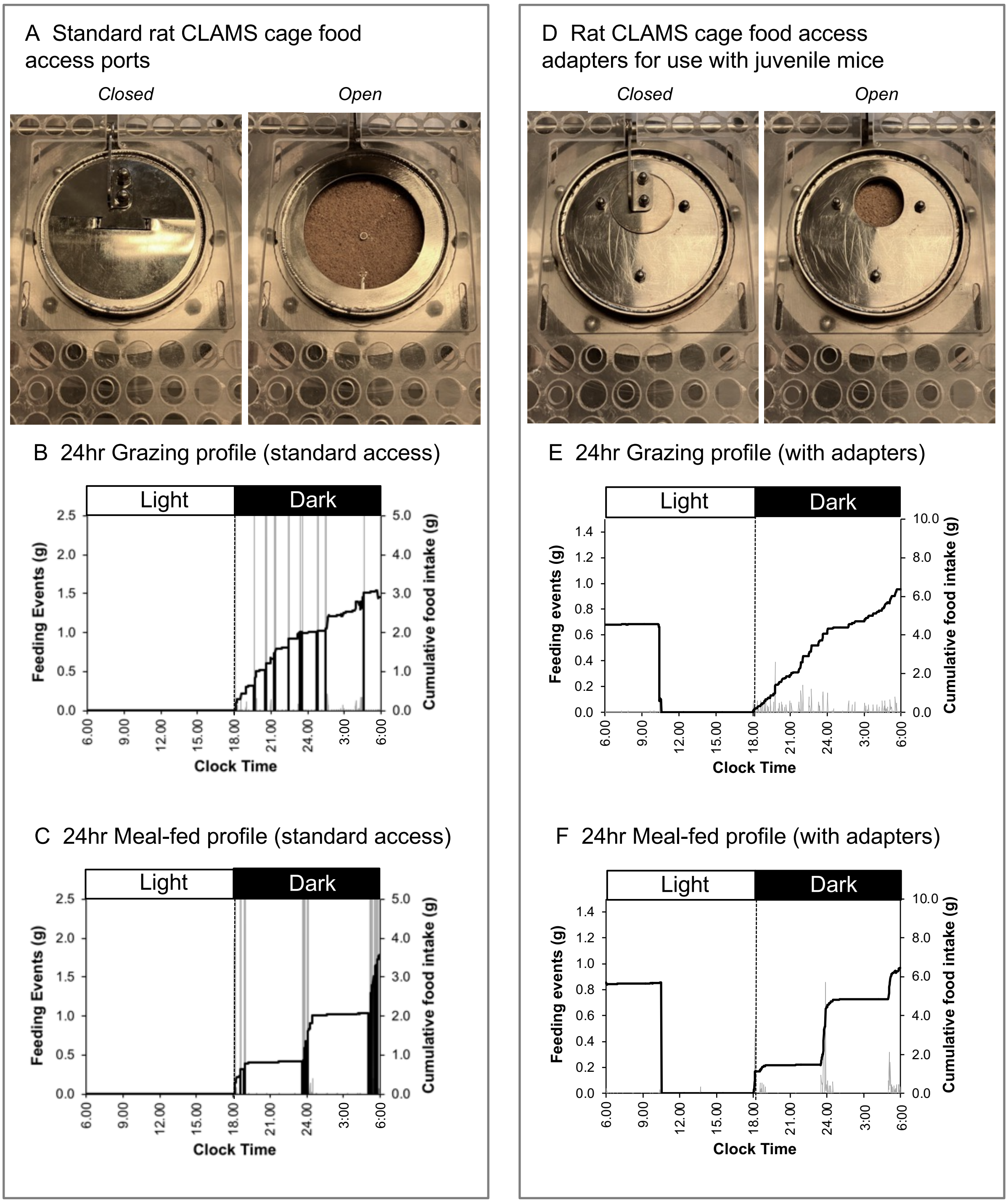

Supplementary Figure S1: CLAMS Food access port adapters improve patterned food delivery profiles in mice (Study 4). Mice placed in the standard rat CLAMS system (**A**) produce large positive and negative deflections in feeding profiles (**B**,**C**). Stainless steel adaptor plates (**D**) remove these deflections in the grazing and meal-fed profiles (**E**,**F**), yielding clear feeding pattern delivery and permitting use of juvenile mice.

Supplementary Table 3: *Post-mortem* analysis of growth end points in pattern-fed 6-week old WT and GHS-R-null mice (Study 4)

	WT			GHS-R-null		
	Ad libitum (n=8)	**Grazing** (n=8)	**Meal-fed** (n=8)	***Ad libitum*** (n=9)	**Grazing** (n=8)	**Meal-fed** (n=9)
Nose-anus length (cm)	9.05 ±0.09	8.96 ±0.08	8.76 ±0.11	8.77 ±0.11	8.78 ±0.14	8.74 ±0.06
Tibial length (mm)	17.11 ±0.06	17.12 ±0.09	16.93 ±0.17	16.94 ±0.11	16.86 ±0.13	16.98 ±0.08
Plasma [IGF-1] (ng/ml)	2600 ±252	2703 ±373	3082 ±693	2186 ±171	1866 ±266	1956 ±276
Pituitary weight (mg)	1.43 ±0.12	1.30 ±0.06	1.18 ±0.11	1.26 ±0.06	1.24 ±0.08	1.06 ±0.05
Liver weight (%-BW)	4.84 ±0.16	4.87 ±0.25	5.09 ±0.21	4.71 ±0.17	4.81 ±0.11	4.99 ±0.25
Kidney weight (%-BW)	0.63 ±0.03	0.62 ±0.03	0.61 ±0.02	0.70 ±0.02	0.64 ±0.02	0.68 ±0.04
Adrenal weight (mg)	2.85 ±0.19	2.80 ±0.22	2.63 ±0.16	2.70 ±0.14	2.69 ±0.25	2.62 ±0.18

Values shown are mean ± SEM

Supplementary Table 4: *Post-mortem* analysis of growth end points in pattern-fed rats (Study 5)

	Ad libitum (n=9)	**Grazing** (n=4)	**Meal-fed** (n=4)
Cumulative caloric intake (kcal)	2020 ± 31	1716 ± 43 [aaaa]	1770 ± 30 [aaa]
Body weight gain (g)	170.2 ± 3.7	157.3 ± 8.3	172.1 ± 5.8
Nose-anus length (cm)	22.1 ± 0.2	22.1 ± 0.4	21.1 ± 0.2 [a]
Tibial length (mm)	38.5 ± 0.4	38.5 ± 0.5	36.9 ± 0.2
Pituitary weight (mg)	11.0 ± 0.6	12.1 ± 0.4	10.1 ± 0.4
Liver weight (%-BW)	4.26 ± 0.10	4.29 ± 0.04	4.75 ± 0.18 [a]
Kidney weight (%-BW)	0.44 ± 0.01	0.42 ± 0.01	0.38 ± 0.01 [aa]

[a] $P<0.05$; [aa] $P<0.01$; [aaa] $P<0.001$; [aaaa] $P<0.0001$ vs *Ad libitum*

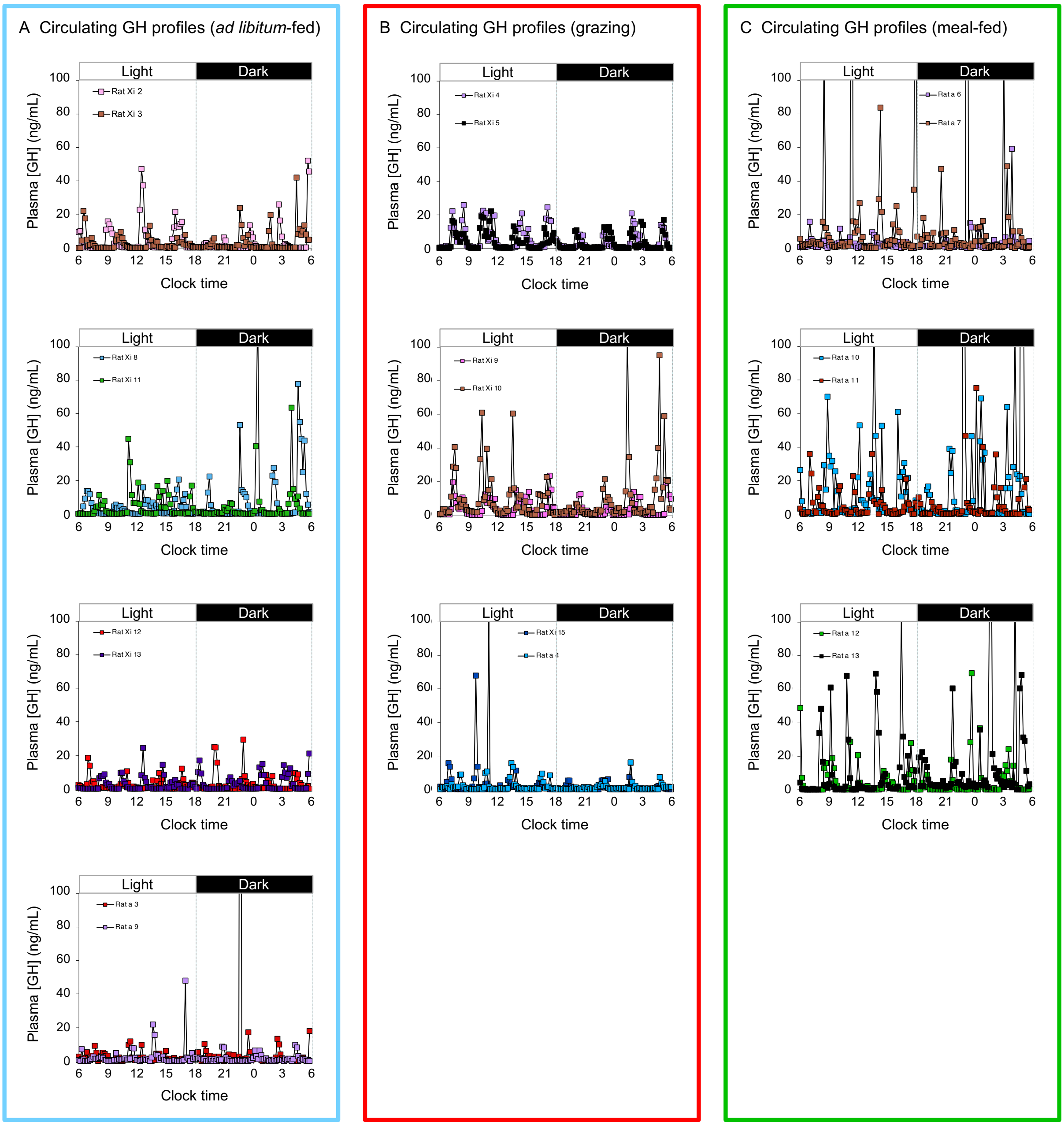

Supplementary Figure S2: Meal-feeding enhances GH secretion and rhythmicity in rats (Study 6). The impact of *ad libitum*-feeding (**A**, blue box), nocturnal grazing (**B**, red box) and nocturnal meal-feeding (**C**, green box) with a standard non-purified rodent diet on individual GH profiles in male rats. Data presented are from pairs of rats (distinguished by colour), with all data shown subjected to detailed profile analysis (Figure 6).

Supplementary Table 5: *Post-mortem* analysis of growth and metabolic end points in pattern-fed rats (Study 6)

	Ad libitum (n=8)	**Grazing** (n=6)	**Meal-fed** (n=6)
Cumulative caloric intake (kcal)	1974 ± 44	1715 ± 44 [aa]	1681 ± 65 [aa]
Body weight gain (g)	171.1 ± 3.4	156.7 ± 3.7	163.3 ± 7.5
Nose-anus length (cm)	21.5 ± 0.2	21.1 ± 0.3	21.2 ± 0.2
Tibial length (mm)	37.7 ± 0.3	37.5 ± 0.4	36.6 ± 0.2
Pituitary weight (mg)	9.8 ± 0.5	9..4 ± 0.3	10.2 ± 0.5
Liver weight (%-BW)	4.45 ± 0.09	4.40 ± 0.20	4.18 ± 0.05
Kidney weight (%-BW)	0.45 ± 0.01	0.45 ± 0.01	0.39 ± 0.01 [aa,bb]

[aa] $P<0.01$ vs *Ad libitum*; [bb] $P<0.01$ vs Grazing

Supplementary Table 6: Human participant characteristics (Study 7)

	Continuously infused (n=8)	**Bolus-infused** (n=8)
Age range (yrs)	18 - 38	19 - 42
Mean age (yrs)	26 ± 3	26 ± 3
Height (m)	1.83 ± 0.03	1.82 ± 0.02
Body Mass Index (kg/m^2)	23.0 ± 0.9	24.1 ± 0.8
Resting metabolic rate (kcal/day)	1866 ± 45	1935 ± 68

Data shown are mean ± SEM
